# 

**Published:** 1875-02

**Authors:** 


					﻿Four Dollars a Year, Postage Free.
Single Copies, 35 Cents.
Vol. XXXII.
February, 1875.
Number 11.
THE
(fcljirflgo	SFonrnfll.
T. ADAMS AT.I.EX, M.D., LL.D., WALTER HAY, M.D.,
EDITORS.
TWELVE TIMES A YEAR.
CHICAGO:
W. B. KEEN, COOKE & CO., Publishers,
Nos. 113 and 115 State Street.
Copyright. A.D. 1875 W. B. KEEN, COOKE & CO.
				

